# Factors Affecting Clinical Course of Postoperative Bile Leakage and Efficacy of Endoscopic Biliary Drainage: A Multi‐Center Retrospective Cohort Study

**DOI:** 10.1002/deo2.70161

**Published:** 2025-06-10

**Authors:** Kota Shimojo, Takuji Iwashita, Keisuke Iwata, Yuki Utakata, Kaori Koide, Takuya Koizumi, Yuki Ito, Yosuke Ohashi, Shota Iwata, Akihiko Senju, Ryuichi Tezuka, Hironao Ichikawa, Yuhei Iwasa, Naoki Mita, Mitsuru Okuno, Kensaku Yoshida, Akinori Maruta, Shinya Uemura, Masahiko Kawai, Yoshiyuki Sasaki, Katsutoshi Murase, Nobuhisa Matsuhashi, Masahito Shimizu

**Affiliations:** ^1^ Department of Gastroenterology Hashima Municipal Hospital Gifu Japan; ^2^ First Department of Internal Medicine Gifu University Hospital Gifu Japan; ^3^ Department of Gastroenterology Gifu Municipal Hospital Gifu Japan; ^4^ Department of Gastroenterology Gifu Prefectural General Medical Center Gifu Japan; ^5^ Department of Surgery Gifu Prefectural General Medical Center Gifu Japan; ^6^ Department of Surgery Gifu Municipal Hospital Gifu Japan; ^7^ Department of Gastroenterological Surgery/Pediatric Surgery Gifu University Hospital Gifu Japan

**Keywords:** bile leakage, endoscopic biliary drainage, intraductal stent placement, postoperative complication, perioperative management

## Abstract

**Introduction:**

Bile leakage is one of the complications after hepatobiliary surgery, causing intra‐abdominal infections, and is sometimes difficult to treat. The purpose of our study was to investigate the factors related to severity and to evaluate the efficacy of endoscopic treatment.

**Methods:**

This was a retrospective multicenter cohort study conducted at three tertiary care medical centers. The severity of bile leakage was classified per the International Study Group of Liver Surgery, and Grades B and C (requiring some intervention or reoperation) were considered as severe.

**Results:**

The subjects were 59 patients. The surgical procedures were 31 cholecystectomies, 23 hepatectomies, and five pancreaticoduodenectomies. The severity was Grade A/B/C: 17/40/2. Multivariate logistic regression analysis found that age (unit odds ratio [UOR], 1.09; 95% confidence interval [CI], 1.0–1.19; *p* = 0.049) and days from surgery to bile leak (UOR, 1.18; 95% CI, 1.04–1.35; *p* = 0.012) were independent predictors of bile leak severity. Of 40 Grade B biliary leakage patients, 37 patients underwent endoscopic drainage, of which 11 also received intra‐abdominal abscess drainage. Eventually, bile leakage was successfully treated in all patients after several endoscopic drainage sessions, and the median drainage period was 18 days (inter‐quartile range: 13–35).

**Conclusion:**

In the management of bile leakage after hepatobiliary surgery, elderly patients or patients with late onset of bile leak may be at high risk of severity. Endoscopic biliary drainage is considered a safe and effective treatment for severe patients.

## 1 Introduction

Bile leakage is the unintended leak of bile from the biliary system or liver and is one of the common complications of hepatobiliary surgery. The incidence of postoperative biliary leakage is reported to be 0.8%–1.4% following laparoscopic cholecystectomy, and 3.6%–12% after hepatectomy without bile duct reconstruction, reducing to 0.4%–8% with bile duct reconstruction, as indicated by previous studies [1, 2, 3, 4]. Bile leakage can lead to intra‐abdominal abscesses, sepsis, prolonged hospital stays, and decreased daily activities [5, 6, 7]. Intraperitoneal drains placed during surgery can offer conservative treatment for bile leakage if drainage is effective. Nevertheless, persistent bile leakage necessitates invasive interventions. Treatments such as endoscopic biliary drainage, percutaneous transhepatic biliary drainage (PTBD), percutaneous intraperitoneal drainage, and surgical approaches have been utilized, but a definitive treatment strategy is yet to be established. Recently, endoscopic transpapillary bile duct drainages, including endoscopic nasobiliary drainage (ENBD) and endoscopic biliary drainage (EBD), have been considered a minimally invasive treatment for bile leakage [8, 9]. However, its efficacy and safety have not been fully investigated, particularly in patients who are more effectively treated with endoscopy, the optimal type of stent to be used, among other considerations. This study aims to evaluate the factors affecting the clinical course of postoperative bile leakage and the efficacy and safety of endoscopic transpapillary bile duct drainages in its management.

## 2 Methods

### 2.1 Patient Selection

This retrospective multicenter cohort study was conducted at Gifu Municipal Hospital, Gifu Prefectural General Medical Center, and Gifu University Hospital between January 2012 and February 2023. Data were extracted from a database. Eligible participants were patients aged 18 years and older who experienced bile leakage following hepatobiliary surgery. We excluded patients whose bile leakage resulted from non‐surgical causes. The Institutional Review Boards of Gifu University Hospital (registration number: 2023–082, date of approval: August 13, 2023) approved this study, which adhered to the tenets of the Declaration of Helsinki. Participant consent was obtained through an opt‐out methodology.

### 2.2 Treatment Strategy

The primary approach for postoperative bile leakage was conservative treatment, provided effective drainage was established via an intraperitoneal drain during surgery. If bile leakage persisted for about a week, or if patients experienced abdominal pain or infection, endoscopic biliary drainage was employed to alleviate intrabiliary pressure. For patients where endoscopic intervention was impractical, such as after gastrointestinal tract reconstruction, PTBD was selected. Intra‐abdominal abscesses and infected biloma were managed by percutaneous or transgastrointestinal abscess drainage as required. Surgical intervention was reserved for patients with intestinal stenosis or colon fistulas complicating intestinal abscesses and deemed difficult to manage without surgical intervention.

### 2.3 Endoscopic Biliary Drainage

Endoscopic retrograde cholangiopancreatography (ERCP) was conducted using side‐viewing endoscopes (JF‐260 V or TJF‐260 V; Olympus, Tokyo, Japan) while using small bowel double‐balloon endoscopes (EN‐580T; Fujifilm, Tokyo, Japan) for surgical altered upper intestinal anatomy. Following bile duct intubation, cholangiography was performed to evaluate bile duct injury and stenosis. A drainage tube was inserted to reduce intrabiliary pressure, and placed at the leak site or as far upstream as possible. Commonly, 5Fr or 6Fr ENBD (Gadelius Medical, Tokyo, Japan, or Piolax Medical, Yokohama, Japan) tubes were used. In patients at risk of ENBD self‐extraction or requiring long‐term placement due to bile duct stricture, EBD was chosen based on the endoscopist's judgment. Stents used included plastic stents (PS, Through & Pass; Gadelius Medical, Tokyo, Japan or Flexima; Boston Scientific, MA, USA) or fully covered self‐expandable metallic stents (FCSEMS, M‐intra; Medicos Hirata, Tokyo, Japan or Niti‐S; Taewoong Medical, Gimpo, Korea). ENBD was removed after clinical improvement, based on the patient's general condition, the amount of drainage through the intraperitoneal drain, and cholangiography findings through ENBD, usually 1–2 weeks after ENBD placement. For EBD, follow‐up ERCP was performed to confirm leak healing and to remove the stent, usually 1–3 months after EBD placement.

### 2.4 Definition

Bile leakage was defined as bile discharge from a postoperative drain (bilirubin concentration in the drain fluid at least thrice the serum bilirubin concentration) or contrast leakage on ERCP [2]. Technical success entailed successful bile duct intubation and drainage catheter placement at the initial endoscopy. Clinical success was the resolution of bile discharge from the drain and the absence of leakage on ERCP or contrast via ENBD. The severity of postoperative bile leakage was classified per the International Study Group of Liver Surgery (ISGLS): Grade A (no treatment required), Grade B (therapeutic intervention excluding reoperation), and Grade C (requiring reoperation), with Grade B and C considered severe [2].

### 2.5 Study Outcomes and Statistical Analysis

Primary endpoints included factors influencing the clinical course of postoperative bile leakage and the clinical success rate of endoscopic treatment. Secondary endpoints encompassed the number of endoscopic interventions, complication rate, length of hospital stay, duration from initial ERCP to discharge, and drainage periods for ENBD and EBD. Continuous variables were expressed as median (interquartile range [IQR]), and the Mann‐Whitney U test was applied to non‐normally distributed continuous variables. Chi‐square tests were used in comparison for categorical variables. Logistic regression analysis was utilized for multivariate analysis between groups to identify possible factors being associated with severe bile leakage, like Grade B or C on ISGLS. Possible factors were age, sex, primary disease, albumin, preoperative cholangitis, drainage, non‐naive papilla, type of surgery, and the interval from surgery to bile leakage onset. A p‐value less than 0.05 was considered statistically significant. Analyses were performed using EZR software version 4.2.1 (Saitama Medical Center, Jichi Medical University, Saitama, Japan).

## 3 Results

This study included 59 patients who experienced bile leakage following hepatobiliary surgery (Gifu Municipal Hospital: 27, Gifu Prefectural General Medical Center: 17, and Gifu University Hospital: 15). The primary disease was benign in 30 patients and malignant in 29 patients. Surgical procedures included cholecystectomy in 31 patients, hepatectomy in 23 patients, and pancreaticoduodenectomy (PD) (child procedure) in five patients. The median value of Alb was 2.7 g/dL. Fifteen patients had preoperative cholangitis, 12 were drained, and 15 were non‐naive papilla. The median duration from surgery to bile leak onset was 3 days. Bile leakage severity was classified as Grade A in 17 patients, Grade B in 40 patients, and Grade C in two patients. Of the Grade B patients (*n* = 40), 37 patients underwent endoscopic biliary drainage, of which 11 also received intra‐abdominal abscess drainage (percutaneous in 10 and transgastrointestinal with endoscopic ultrasonography in one), one patient required PTBD due to endoscopic approach challenging, and two patients received only percutaneous abscess drainage. The two patients in Grade C underwent reoperation (Table 1).

**TABLE 1:** Clinical characteristics.

Bile leakage (*n* = 59)	
Age, years, median (IQR)	72 (68–78)
Male, *n* (%)	36 (61)
Disease, *n* (%)	
Benign	30 (51)
Cholecystitis	23
Cholecystolithiasis	6
Intrahepatic stone	1
Malignant	29 (49)
Metastatic liver cancer	8
Bile duct cancer	8
Hepatocellular cancer	7
Gallbladder cancer	3
Pancreatic cancer	2
Papillary cancer	1
Albumin (g/dL), median (IQR)	2.7 (2.4–3.3)
Preoperative cholangitis, *n* (%)	15 (25)
Drainage, *n* (%)	12 (20)
Non‐naive papilla, *n* (%)	15 (20)
Surgical procedure, *n* (%)	
CCE	31 (53)
HT	23 (39)
Subsegmentectomy	7
Left lobectomy	6
Limited section	5
Segmentectomy	4
Right lobectomy	1
PD	5 (8)
Severity, *n* (%)	
Grade A	17 (29)
Grade B	40 (68)
Grade C	2 (3)
Treatment, *n* (%)	
Conservative	17 (29)
Endoscopic biliary drainage	37 (63)
※Combine with abscess drainage in 11	
Transhepatic biliary drainage	1 (2)
Percutaneous abscess drainage	2 (3)
Re‐operation	2 (3)
Surgery to Bile leak onset (day), median (IQR)	3 (1‐13)

Abbreviations: CCE, cholecystectomy; HT, hepatectomy; PD, pancreaticoduodenectomy.

Multivariate logistic regression analysis was utilized to examine factors influencing the severity of postoperative bile leakage. Patient‐related factors included age, sex, primary disease, albumin, preoperative cholangitis, drainage, and non‐naive papilla; intraoperative factors included surgical procedures; postoperative factors encompassed the interval from surgery to bile leak onset. Age (unit odds ratio [UOR], 1.09; 95% confidence interval [CI], 1.0–1.19; *p* = 0.049) and the interval from surgery to bile leak onset (UOR, 1.18; 95% CI, 1.04–1.35; *p* = 0.012) were independent predictors of bile leak severity (Table 2).

**TABLE 2:** Result of multivariate analysis to identify predictors of bile leak severity.

			Univariate analysis		Multivariate analysis	
	Grade A *n* = 17	Grade B, C *n* = 42	OR (95% CI)	*p*‐Value	OR (95% CI)	*p*‐Value
Age, years, median (IQR)	70 (68–71)	74 (68–78)	1.06(Unit) (0.99–1.13)	0.087	1.09 (Unit) (1.0–1.19)	0.049
Sex, Male/Female, *n*	13/4	23/19	0.37 (0.1–1.33)	0.12	2.39 (0.43–13.1)	0.31
Disease, Malignant/Benign, *n*	13/4	16/26	0.18 (0.05–0.68)	0.011	0.175 (0.02–1.47)	0.10
Albumin, g/dL median (IQR)	2.7 (2.4–3.2)	2.8 (2.5–3.3)	1.37(Unit) (0.52–3.58)	0.98		
Preoperative cholangitis Yes/No, *n*	5/12	10/32	0.75 (0.21–2.65)	0.65		
Drainage Yes/No, *n*	4/13	8/34	0.76 (0.76–2.98)	0.69		
Non‐naive papilla Yes/No, *n*	5/12	10/32	0.75 (0.21–2.65)	0.65		
Surgical procedure, HT/CCE and PD, *n*	11/6	12/30	0.21 (0.06–0.72)	0.012	0.295 (0.04–2.14)	0.22
Surgery to leak, days, median (IQR)	3 (1–6)	5 (1–13)	1.09(Unit) (0.98–1.2)	0.096	1.18 (Unit) (1.04–1.35)	0.012

OR, Odds ratio; CI, Confidence interval; IQR, interquartile range; CCE, Cholecystectomy;.

HT, Hepatectomy; PD, Pancreaticoduodenectomy.

**FIGURE 1:**
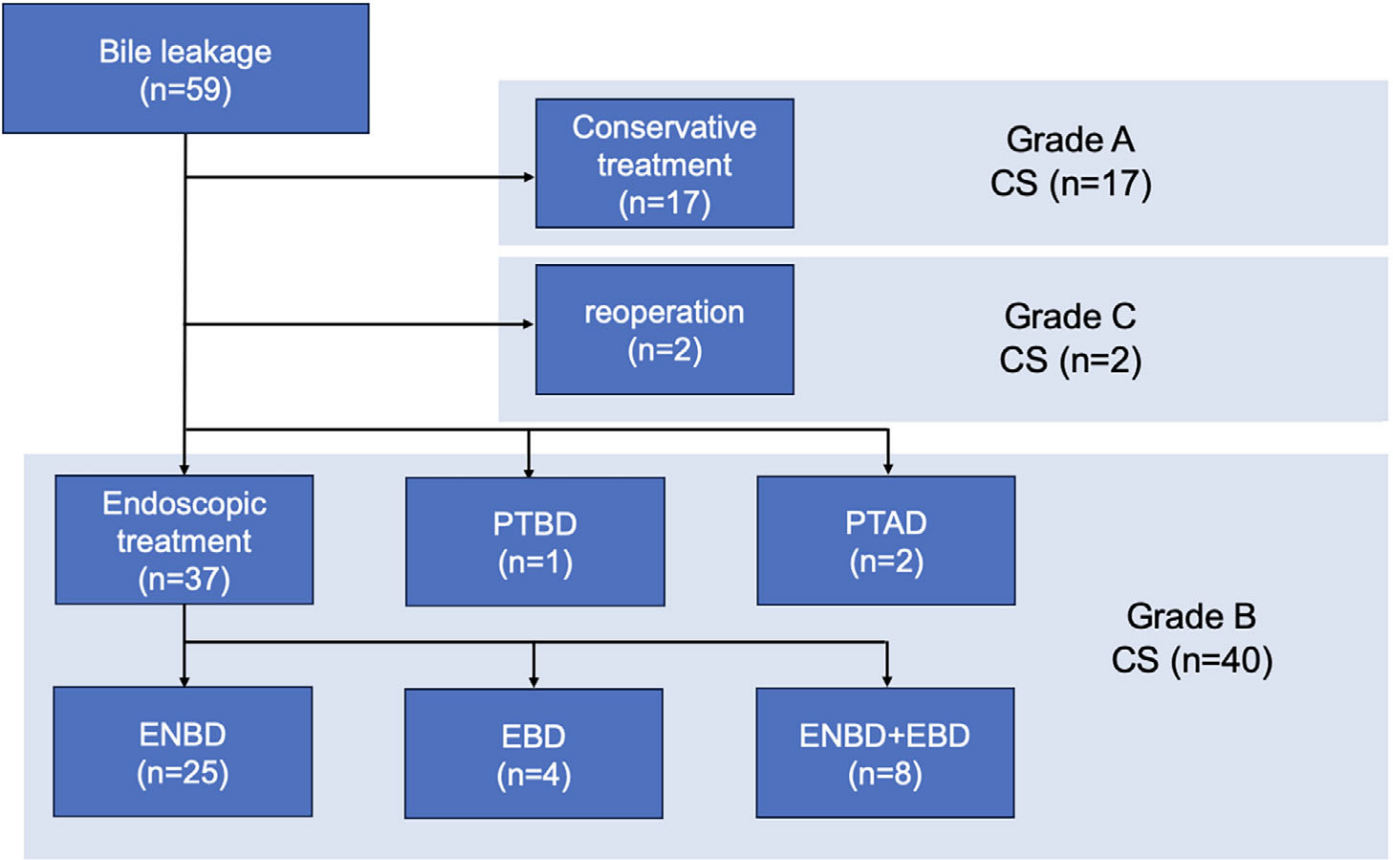
Flow of the study patients (Original figure). ENBD: endoscopic naso‐biliary drainage, EBD: endoscopic biliary drainage, PTBD: percutaneous transhepatic biliary drainage, PTAD: percutaneous abscess drainage, and CS: clinical success.

ERCP was successful in all 37 patients who underwent endoscopic biliary drainage, with bile leakage confirmed in 32 patients, all of the non‐disrupted types. Leakage locations were as follows: 15 from the cystic duct, seven from the intrahepatic bile duct, four from both right and left hepatic ducts, three from the common bile duct, and three from choledochojejunostomy. Of these, the drainage was performed across the leakage locations in 25 patients. During the initial ERCP, ENBD was placed in 31 patients and EBD in six patients (PS in four patients and FCSEMS in two patients), achieving a technical success rate of 100%. Of these, 29 patients were resolved after initial endoscopic drainage. Combined drainage of ENBD and EBD was performed in eight patients because of confirmed minor bile leakage in three, accidental ENBD removal in two, spontaneous ENBD migration in one, stricture of choledochojejunostomy in one, and stricture of the injured bile duct in one. The median number of endoscopies performed was 1 (range: 1–4), and the median duration between the first and second sessions was 11 days (range: 4–33). Ultimately, 25 patients were resolved with ENBD, four with EBD, and eight with a combination of both (shown in Figure 1).

Eventually, bile leakage was successfully treated in all patients after several endoscopic drainage sessions, resulting in a clinical success rate of 100%. The complication rate of the endoscopic procedure was 8% (3/37), including spontaneous stent migration using a straight type stent in two and biliary bleeding caused by pseudoaneurysm by SEMS in one. Stent migrations were managed by endoscopic re‐treatment. The patients with biliary bleeding underwent long‐term (2 months) FCSEMS placement and required angiography to manage it. The median hospital stay was 33 days, with the median interval from initial ERCP to discharge being 26 days, and the median drainage period being 18 days (Table 3).

**TABLE 3:** Outcomes of endoscopic biliary drainage.

Endoscopic drainage (*n* = 37)	
Drainage methods	
ENBD, *n* (%)	25 (67)
ENBD+EBD, *n* (%)	8 (22)
EBD, *n* (%)	4 (11)
Number of endoscopy interventions, median (range)	1 (1–4)
Technical success, *n* (%)	37/37 (100)
Clinical success, *n* (%)	37/37 (100)
Complication, *n* (%)	3/37 (8)
Hospital stay, days, median (IQR)	33 (23–49)
ERCP to discharge, days, median (IQR)	26 (16–33)
Drainage period, days, median (IQR)	18 (13–35)

Abbreviations: EBD endoscopic biliary drainage; ENBD, endoscopic nasobiliary drainage; IQR, interquartile range.

**FIGURE 2:**
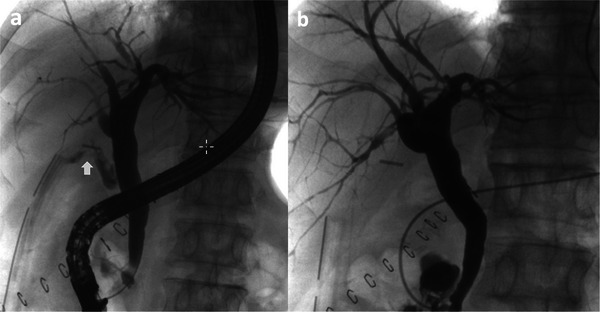
Bile leakage treated with ENBD (Original figure). A 70‐year‐old male underwent cholecystectomy for acute cholecystitis. Bile leakage from the cystic duct (Arrow) on 1 postoperative day (POD) was confirmed by ERCP and ENBD was then placed (a). Cholangiography through ENBD on 9 POD confirmed no leakage and ENBD was removed. (b). POD: postoperative day, ERCP: endoscopic retrograde cholangiopancreatography, and ENBD: endoscopic naso‐biliary drainage.

**FIGURE 3:**
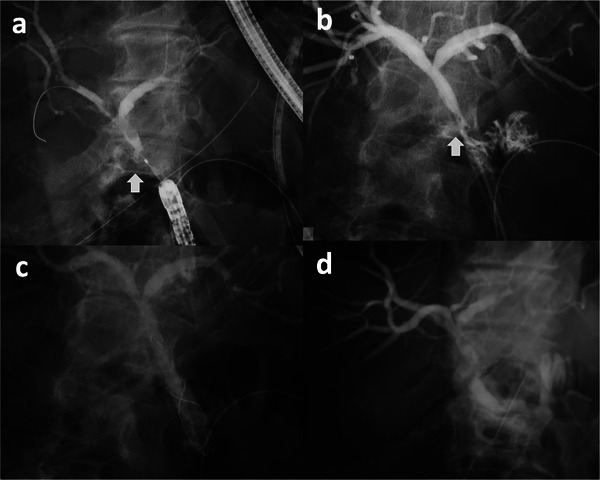
Bile leakage treated with ENBD and EBD (Original figure). A 59‐year‐old female who underwent pancreatoduodenectomy for pancreatic cancer had bile discharge from the intraperitoneal drain during surgery on 1 POD. Bile discharge persisted and the inflammatory reaction worsened on 9 POD, so she underwent ERCP. ERCP confirmed contrast extravasation from choledochojejunostomy (arrow) (a) and ENBD was then placed (b). Cholangiography through ENBD showed persistent contrast leakage and anastomotic stricture (arrow) on 21 POD and FCSEMS was then placed to cover the leak site (c). Follow‐up ERCP performed 3 months later confirmed no bile leakage (d). POD: postoperative day, ERCP: endoscopic retrograde cholangiopancreatography, ENBD: endoscopic naso‐biliary drainage, and FCSEMS: fully covered self‐expandable metallic stent.

The median follow‐up period was 58.8 months, with no recurrences observed. A patient treated solely with ENBD is illustrated in Figure 2, and a patient treated with both ENBD and EBD is depicted in Figure 3.

When categorized into two groups—one receiving ENBD as the initial endoscopic treatment and the other EBD—the clinical success rates were 77.4% and 83.3%, respectively (*p* = 1.0). No significant differences were observed in the number of endoscopic interventions, complication rates, hospital stays, periods from initial ERCP to discharge, or drainage periods (Table 4).

**TABLE 4:** Outcomes of initial endoscopic drainage for endoscopic nasobiliary drainage (ENBD) and endoscopic biliary drainage (EBD).

	ENBD, *n* = 31	EBD, *n* = 6	*p*‐Value
Clinical success, *n* (%)	24/31 (77.4)	5/6 (83.3)	1
Complication, *n* (%)	2/31 (6.5)	1/6 (16.7)	0.42
No. of endoscopic intervention, median (range)	1 (1‐4)	1 (1‐4)	0.86
Hospital stay, days, median (IQR)	33 (24.5–43)	39 (22–53)	0.98
ERCP to discharge, days, median (IQR)	26 (17–31.5)	21 (6.5–43.5)	0.64
Drainage period, days, median (IQR)	17 (12–28.5)	72 (61.5–82.5)	0.09

Abbreviations: EBD endoscopic biliary drainage; ENBD, endoscopic nasobiliary drainage; IQR, interquartile range.

## 4 Discussion

This research evaluated the factors associated with the severity of biliary leakage and the effectiveness of endoscopic intervention. Advanced age and the duration between surgical procedures and the onset of bile leakage were identified as independent predictors for more severe bile leaks. In a cohort of 40 patients diagnosed with Grade B biliary leakage, endoscopic drainage was administered to 37 patients. The procedure successfully resolved bile leakage in all treated patients.

When conservative treatment for bile leakage fails, therapeutic intervention becomes essential [10]. A previous study indicated that bile leaks occurring over 2 weeks post‐surgery are associated with increased severity compared to those occurring earlier [10]. Our study also identified an increased duration from surgery to bile leak detection as a risk factor for severity. This disparity in pathogenesis may be attributed to early onset leaks resulting from intraoperative bile duct injury, liver dissection, or suture failure of the bile duct or cystic duct. In contrast, delayed onset bile leaks often arise from bile duct wall failure due to intraperitoneal infection. Additionally, delayed detection and treatment of late‐onset patients, often due to the removal of operative drains, can lead to complications such as intra‐abdominal abscess or sepsis [10]. These findings underscore the possible association between the increased risk of developing severe bile leak and the late onset of that.

Endoscopic biliary drainage is indicated when conservative treatments prove ineffective. Recent literature underscores its efficacy [8, 9, 11–18], and our study corroborates these findings with a 100% clinical success rate. ERCP facilitates direct visualization of contrast extravasation, allowing precise identification of the leakage site and evaluation of potential impediments to healing, such as bile duct stones or strictures [19]. Post‐diagnosis, treatment options include ENBD, EBD, or a combination thereof. Theoretically, endoscopic transpapillary biliary drainage aids in healing bile leaks by diminishing the transpapillary pressure gradient and redirecting bile flow toward the ampulla [8, 9]. While both ENBD and EBD demonstrate favorable outcomes, consensus on the optimal endoscopic intervention remains elusive. In this study, ENBD was basically selected due to its benefits in draining the bile duct with a greater pressure gradient than internal drainage, monitoring bile discharge, and facilitating leak healing assessment via cholangiogram. However, prolonged use of ENBD may detract from quality of life and elevate the risk of self‐extraction by patients [20, 21]. Conversely, EBD, while not interfering with daily activities and devoid of self‐extraction risks, lacks the capability for monitoring or conducting cholangiography. In our study, EBD was mainly chosen for elderly patients who had a higher risk for self‐removal of ENBD and for patients with biliary strictures requiring stent placement.

Regarding EBD placement, the choice between PS and FCSEMS is debated, with insufficient research on the optimal stent type. Previous studies have reported PS to be effective, cost‐efficient, safe, and easily removable [9, 22]. In contrast, FCSEMS, although more costly and challenging to remove compared to PS, demonstrates superiority in patients with persistent bile leakage over multiple PS placements [23]. FCSEMS significantly reduces the transpapillary pressure gradient due to its larger diameter and can directly cover the leak site, by matching the diameter of the damaged bile duct. In our study, PS was predominantly used for EBD, with FCSEMS initially chosen in two patients where extensive bile duct damage was anticipated, potentially leading to refractory leaks [24]. This approach resulted in successful resolution in all instances but ultimately required multiple endoscopic procedures to resolve bile leakage. Regarding complications, one patient with FCSEMS developed a pseudoaneurysm two months post‐stenting, causing biliary bleeding that was resolved with transcatheter arterial embolization. These adverse events, associated with prolonged stenting [9], underscore the need for caution regarding indications for FCSEMS and the duration of stent placement.

The optimal duration for drainage remains to be conclusively determined. In patients involving ENBD placement, cholangiography is typically recommended 1–2 weeks after placement. Following this, the ENBD is removed once the healing of the leak is confirmed. Early cholangiography is generally discouraged due to the risk of exacerbating the leak site, especially when it is close to closure [25]. In terms of PS, the European Society of Gastrointestinal Endoscopy suggests a placement duration ranging from 4 to 8 weeks [26]. Originally, it was proposed that FCSEMS could be removed within 3 months [27]. However, more recent studies advocate for a shorter duration of 3–4 weeks to reduce the risk of complications [9, 28]. In our research, patients with ENBD underwent cholangiography about 1 week following placement. Patients with EBD received a follow‐up ERCP between 1 and 3 months post‐placement to evaluate the resolution of the leak. Drainage tubes were extracted once contrast extravasation ceased, with the median drainage period being 18 days. Based on these findings, the timing for reevaluation of a bile leak could be reasonably set at 1 week for ENBD and 1 month for EBD.

Endoscopic transpapillary drainage, while advantageous in managing bile leaks, exhibits limitations. Its efficacy is reduced in scenarios where significant bile accumulation occurs in the abdominal cavity or when an intraperitoneal abscess has formed [29, 30]. In our study cohort, out of 37 patients who underwent endoscopic treatment, 11 required additional abscess drainage (10 via a percutaneous approach and one through a transgastrointestinal method with endoscopic ultrasonography). It also presents challenges following a gastrointestinal reconstruction or in instances involving intestinal strictures. Furthermore, patients with bile leakage of disrupted type, which lack a connection to the bile duct, are difficult to manage by endoscopic drainage. Although a standard treatment for the disrupted bile duct has not been established, those patients typically required percutaneous interventions such as placement of a drainage catheter and intrahepatic biliary ablation [31]. PTBD is also underscored its efficacy by some literature [32], and has advantages, such as the possibility of treating patients for whom endoscopic treatment is not feasible or effective for any reason. The usefulness of intrahepatic biliary ablation with ethanol for refractory bile leakage has also been reported [33]. Therefore, the decision to employ endoscopic treatment, in conjunction with alternative drainage methods, must be made with careful consideration. This decision should be made based on a comprehensive assessment of the patient's overall condition, including the extent of bile leakage, the presence of intra‐abdominal abscess complications, any prior gastrointestinal reconstruction, and the presence of intestinal strictures.

There might be several limitations to this study. The retrospective nature of this study resulted in a lack of standardized protocol for patient and stenting duration or selection, leading to potential bias in these selections. Additionally, the small sample size has made it difficult to further subdivide analysis in the type of hepatectomy for risk of severity, which may limit the statistical robustness of the findings.

## 5 Conclusion

In the management of bile leakage after hepatobiliary surgery, elderly patients, as well as those experiencing delayed onset of bile leakage, may possess an elevated risk of severe complications. Endoscopic biliary drainage could be a safe and effective intervention for the severe status of this complication. While both ENBD and EBD demonstrate favorable outcomes, the choice of drainage method should be based on the individual patient's clinical condition. However, alternative drainage methods, such as percutaneous drainage, should be combined with endoscopic one, if endoscopic drainage is not effective. Further evaluation is required with a prospective design and larger cohort to confirm these findings.

## Conflicts of Interest

The authors declare no conflicts of interest.

## Ethics Statement

**Approval of the research protocol by an Institutional Reviewer Board**: Gifu University Hospital, approval number 2023–082, date of decision August 13, 2023.

## Consent

Informed Consent was obtained in the form of opt‐out.

## Clinical Trial Registration

N/A
